# 

**Published:** 1949-01

**Authors:** 


PUBLISHED UNDER THE AUSPICES OF THE BRISTOL MEDICO-CHIRURGICAL SOCIETY

### THE BRISTOL MEDICO-CHIRURGICAL JOURNAL

*A Journal of the Medical Sciences for the*

### WEST OF ENGLAND AND SOUTH WALES

*Editor:*
E. WATSON-WILLIAMS, *M.C.*, M.D., Ch.M., F.R.C.S.

*with whom are associated*
G. E. F. SUTTON, *M.C.*, M.D., F.R.C.P.
W. MELVILLE CAPPER, M.B., B.S., F.R.C.S.
J. M. NAISH, M.D., M.R.C.P.
N. S. CRAIG, *M.C.*, M.D., *Assistant Editor.*

*Local Correspondents:*

BATH—A. D. BATEMAN, *O.B.E.*, F.R.C.S. CARDIFF—J. D. SPILLANE, M.D., M.R.C.P.

EXETER—Dr. L. N. JACKSON, *M.C.* GLOUCESTER—Dr. R. F. JARRETT, M.B., M.R.C.P.

NEWPORT—J. L. D. WILLIAMS, M.B., F.R.C.S. PLYMOUTH—Dr. C. R. CROFT.

TAUNTON—Dr. M. McALLEY, D.M.R.

TORQUAY—Dr. A. ROBINSON THOMAS, D.M.R.E.

TRURO—Dr. F. D. M. HOCKING, F.R.I.C.

BRISTOL:
J. W. ARROWSMITH LTD., QUAY STREET AND SMALL STREET

Vol. LXVI. No. 237. *Price:* FOUR SHILLINGS net. JANUARY · 1949

